# Supplementary material for: Muscle contractile exercise through a belt electrode device prevents myofiber atrophy, muscle contracture, and muscular pain in immobilized rat gastrocnemius muscle
Source: PLoS One. 2022 Sep 23;17(9):e0275175. doi: 10.1371/journal.pone.0275175 (PMC9506634; doi:10.1371/journal.pone.0275175)
Supplement: Attachment — Submitted filename: response letter.docx [file pone.0275175.s002.docx]

**Response to the Editor-in-Chief and reviewers**

We would like to thank both the reviewers for evaluating our study and their constructive criticism, which allowed us to strengthen and clarify our study's conclusions. We have responded in detail to each comment and discussed how the concerns raised were addressed in the revised manuscript.

**Journal Requirements**

***1. Please ensure that your manuscript meets PLOS ONE's style requirements, including those for file naming. The PLOS ONE style templates can be found at https://journals.plos.org/plosone/s/file?id=wjVg/PLOSOne_formatting_sample_main_body.pdf and https://journals.plos.org/plosone/s/file?id=ba62/PLOSOne_formatting_ sample_title_authors_affiliations.pdf***

We revised our manuscript according to The PLOS ONE style templates.

**Comments to the Author**

***3. Have the authors made all data underlying the findings in their manuscript fully available?***

***The PLOS Data policy requires authors to make all data underlying the findings described in their manuscript fully available without restriction, with rare exception (please refer to the Data Availability Statement in the manuscript PDF file). The data should be provided as part of the manuscript or its supporting information, or deposited to a public repository. For example, in addition to summary statistics, the data points behind means, medians and variance measures should be available. If there are restrictions on publicly sharing data—e.g. participant privacy or use of data from a third party—those must be specified.***

We submitted all data sheets (supplemental file) according to your advice.

**Reviewer: 1**

***1. The name of the abscissa axis in Figure 1. B is wrong, and the author wants to express the stimulation time, not the stimulation intensity.***

We revised Fig 1.B according to your advice.

***2. The special symbols for p-values denoting between-group differences in Figure 2. B and Figure 5. B do not exactly match the legends.***

We modified our legends according to your advice.

***3. According to the author's experimental results in Figure 4. A, we found that there are only type IIb muscle fibers in the superficial gastrocnemius muscle in this experiment. How to determine that there are only type IIb muscle fibers in the superficial gastrocnemius muscle?***

We confirmed all parts in the ATPase-stained cross-sections, and we examined the deep regions (supplement figure 1, blue square) and the superficial regions (supplement figure 1, red square) separately. From supplement figure 1, the superficial regions consisted entirely of type IIb fibers. In addition, the previous report supported this point^1)^.

1) Kernell D, Lind A, van Diemen AB, De Haan A. Relative degree of stimulation-evoked glycogen degradation in muscle fibres of different type in rat gastrocnemius. J Physiol. 1995;484:139-153.

***4. In the text, the baseline PPT of the gastrocnemius muscles in the LF group was higher than that in the immobilization group. However, in Figure 6. A, the gastrocnemius muscle baseline PPT value in the LF group was lower than in the fixed group.***

We retouched the figure of PPT according to your advice.

**Reviewer: 2**

**Major essential changes needed:**

***1. In this study, myonuclear apoptosis is mentioned as one of the mechanisms of skeletal muscle atrophy, but the involvement of muscle protein synthesis and degradation is ignored. The total and phosphorylated forms of Akt, p70S6K, and 4E-BP1 (FoxO 1/3 phosphorylation would also be informative) in each experimental group should be added as items to be examined. For example, the time course of the acute effect of a single bout of muscle contractile exercise through the belt electrode device on their phosphorylation should be investigated. In particular, Akt-mTOR signaling pathways may orchestrate both a positive protein metabolism in skeletal muscle and myonuclear apoptosis.***

Your advice was very important. Thomason DB indicated that muscle protein degradation was more influential than muscle protein synthesis for muscle atrophy in 2-week immobilization^1)^. Atrogin-1 and muscle RING-finger protein (MuRF)-1 as ubiquitin-proteasome pathway enzymes played a main role in muscle protein degradation^2)^. Also, peroxisome proliferator-activated receptor gamma coactivator (PGC)-1α was the main regulator for Atrogin-1 and MuRF-1^3)^. Therefore, we examined the mRNA expression of PGC-1α, Atrogin-1, and MuRF-1. As a result, the PGC-1α mRNA expression in the immobilization and LF groups was significantly lower than that in the control group, that in the control and HF groups was no significant difference. Additionally, the Atrogin-1 and MuRF-1 mRNA expressions in the experimental groups were significantly higher than those in the control group, whereas those in the HF group were significantly lower than those in the immobilization and LF groups. From these results, muscle contractile exercise through a belt electrode device may prevent the progression of muscle protein degradation.

These contents were reflected in our manuscript.

1) Thomason DB, Booth FW. Atrophy of the soleus muscle by hindlimb unweighting. J Appl Physiol (1985). 1990;68:1-12.
2) Bodine SC, Latres E, Baumhueter S, Lai VK, Nunez L, Clarke BA et al. Identification of ubiquitin ligases required for skeletal muscle atrophy. J Science. 2001;294:1704-1708.
3) Kang C, Ji LL. Muscle immobilization and remobilization downregulates PGC-1α signaling and the mitochondrial biogenesis pathway. J Appl Physiol 2013;115: 1618-1625.

***2. Methods. In statistical analysis for "range of motion on ankle joint dorsiflexion" and "pressure pain threshold", a split-plot ANOVA using 2 factors (time [baseline vs. 1W vs. 2W] and group [control vs. immobilization vs. LF vs. HF]) must be used to determine the interaction and main effects, after verifying the normality of the data.***

Thank you for your advice. We modified the statistical process. "range of motion on ankle joint dorsiflexion" and "pressure pain threshold" were assessed using two-way ANOVA, followed by Scheffé’s method. Differences were considered statistically significant at p < 0.05. We added these contents to our manuscript (P10, L218-219).

**Minor essential changes needed:**

***1. Methods. Please add the length of time between last electrical stimulation, and euthanasia and tissue collection. Exercise itself can raise macrophage accumulation, NGF upregulation, and some cytokines in tissues. This will help exercise physiology readers interpret the results compared to their own.***

Based on the previous study, we biopsied 12 hours after the last electrical stimulation^1)^. We added this contents to our manuscript (P8, L169-170).

1) Tanaka M, Morifuji T, Sugimoto K, Akasaka H, Fujimoto T, Yoshikawa M, et al. Effects of combined treatment with blood flow restriction and low-current electrical stimulation on capillary regression in the soleus muscle of diabetic rats. J Appl Physiol (1985). 2021:1219-1229.

***2. Methods. The authors described "2 sec (do)/6 sec (rest) duty cycle" as low-frequency and "2 sec (do)/2 sec (rest) duty cycle" as high-frequency for electrical stimulation conditions. It is necessary to clarify the definition of low-frequency or high-frequency in the electrical stimulation conditions.***

Dow DE examined the effect of the difference in the number of muscle contractions on muscle atrophy^1)^. Dow DE suggested that 200 contractions per day would be a good design choice to maintain mass, force, and ﬁber CSA while minimizing energy transfer that may negatively affect the tissue and decrease battery life. In our study, the LF and HF groups had 150 and 225 contractions per day. Therefore, we distinguished the 2 groups from the number of muscle contractions.

1) Dow DE, Cederna PS, Hassett CA, Kostrominova TY, Faulkner JA, Dennis RG. Number of contractions to maintain mass and force of a denervated rat muscle. Muscle Nerve. 2004;30:77-86.

***3. Methods. To mention that 60% maximal voluntary contraction is most effective in preventing skeletal muscle atrophy, should be provided a clear link to the previous studies referenced. In general, it is defined not only by the intensity of muscle contraction exercise, but also by the number of exercises and the number of sets.***

American College of Sports Medicine indicated that the intensity of muscle contractile exercise was one of the important factors for increasing muscle strength^1)^. Concretely, the muscle contractile exercise in 60% MVC produced the largest effect sizes for strength increases^1)^. Furthermore, the same report suggested that the alteration of cross-sectional areas was one of the main components to define muscle strength^1)^. From these, we surmised that the muscle contractile exercise in 60% MVC was most effective to prevent muscle atrophy.

1) American College of Sports Medicine. American College of Sports Medicine position stand. Progression models in resistance training for healthy adults. Med Sci Sports Exerc. 2009;41:687-708.

***4. Methods. What is the stimulus intensity you set when you conducted the preliminary experiment to determine the stimulus time?***

We set the stimulus intensity to 4.7 mA in the preliminary experiment to determine the stimulus time.

***5. Results. For histological observation, the quality of the tissue images stained with immunohistochemistry (Figure 2A) and H&E (Figure 3) were poor in the PDF file.***

We modified the dpi of figure 2A and 3.

**Minor suggested changes:**

***1. Introduction. Hypotheses should be added at the end of the Introductory section.***

We revised our manuscript according to your advice.

***2. Results. In lines 224 on page 10 to line 226 on page 11, "In the immobilization, LF, and HF groups..." seems to be more appropriate than "In the immobilization, LF, HF groups...".***

We modified our manuscript according to your advice (P11, L225).

***3. Results. In lines 253-254 on page 12, “The CSA of type IIa myofibers was...” could be more accurate if change to “The CSA of type IIa myofibers in the deep region was...”.***

We retouched our manuscript according to your advice (P12, L254 and L257).
